# Health literacy of informal caregivers of older adults with dementia: results from a cross-sectional study conducted in Florence (Italy)

**DOI:** 10.1007/s40520-022-02271-0

**Published:** 2022-10-19

**Authors:** Chiara Lorini, Primo Buscemi, Enrico Mossello, Annamaria Schirripa, Barbara Giammarco, Lisa Rigon, Giuseppe Albora, Duccio Giorgetti, Massimiliano Alberto Biamonte, Letizia Fattorini, Rita Manuela Bruno, Gemma Giusti, Yari Longobucco, Andrea Ungar, Guglielmo Bonaccorsi

**Affiliations:** 1 Department of Health Science, University of Florence, Florence, Italy; 2 Health Literacy Laboratory, Department of Health Science, University of Florence, Florence, Italy; 3 Medical Specialization School in Hygiene and Preventive Medicine, University of Florence, viale GB Morgagni 48, 50134 Florence, Italy; 4 Division of Geriatric and Intensive Care Medicine, Careggi Hospital, Firenze, Italy; 5 School of Geriatric Medicine, Department of Experimental and Clinical Medicine, University of Florence, Florence, Italy; 6 Department of Experimental and Clinical Medicine, University of Florence, Florence, Italy

**Keywords:** Health literacy, Family caregiver, Older adults, Dementia

## Abstract

**Aim:**

The aim of this study was to measure the prevalence of inadequate health literacy (HL) in a sample of family caregivers of older adults with dementia, and to assess the relationship of HL with characteristics of caregiver and persons with dementia.

**Methods:**

The study followed a cross-sectional design. Persons with dementia and their family caregivers were enrolled in an outpatients’ geriatric memory clinic. For the caregivers, the following information was collected: socio-demographic data, level of HL, cognitive impairment (using the Mini-Cog). For persons with dementia, the following data were collected: socio-demographic data, functional status (using the Basic and Instrumental Activities of Daily Living), cognitive impairment (using the Mini Mental State Evaluation, and the Global Deterioration Scale) behavioral and psychological symptoms associated with dementia (assessed using the Neuropsychiatric Inventory).

**Results:**

A total of 174 person with dementia/caregiver dyads were enrolled. About 45% of the caregivers presented a possibility or a high likelihood of inadequate HL. The percentage of caregivers with inadequate HL was higher among spousal caregivers than in offspring. Female gender, higher age and lower education were independent predictors of low HL. On multiple logistic regression analysis, persons with dementia assisted by caregivers with a high likelihood of limited HL presented higher risk of a more severe disease.

**Conclusion:**

The results of this study suggest that the HL of dementia caregivers has to be included in the comprehensive geriatric assessment, to develop an appropriate individualized care plan. Moreover, public health interventions are needed to increase the HL of dementia caregivers.

**Supplementary Information:**

The online version contains supplementary material available at 10.1007/s40520-022-02271-0.

## Introduction

The rise in the number of older people accompanied by the global epidemic of chronic disease has determined a higher burden of care dependency. Predictions for the future show that a great part of older population will need, in the last years of their lives, at least one person in charge for care and assistance [1]. Dementia represents one of the greatest contributors to the reduction of healthy life years as disability is one of its defining features: 55 million adults were affected by dementia in 2019 around the world, and by 2050 they are predicted to reach about 139 million [2].

The need for healthcare of older subjects is a public health priority. In Italy most older people with disability are assisted at home, with limited support by the long-term care system, and high support by formal or informal caregivers [3, 4]. Informal caregivers are unpaid individuals, such as neighbors, family members or friends who provide care, while formal caregivers are defined as paid employees or volunteers from a service provider who provide care in a formal setting, such as a nursing home or assisted living facility [5]. As the number of older people with disability increases, the potential number of caregivers is going to increase as well [6].

The caregiver supports the older person in personal care and instrumental activities of daily living, including health behaviors, such as preparation of adequate meals, use of medications and promotion of safe physical activity [7]. To perform these tasks, a caregiver should be able to have access to the information related to patient’s health and understand it, to establish a valuable communication with both the patient and the healthcare providers, and to be able to manage the services offered by the healthcare system [8, 9]. This is particularly crucial for people with dementia, which typically rely on their caregivers for health issues management, due to cognitive impairment inherent with the disease, and often show behavior disorders that pose them at risk of adverse events of whom they may not be aware. The ability to cope with these tasks depends, for both formal and informal caregivers, particularly on the caregiver’s health literacy (HL), defined as the set of cognitive and social abilities needed to have access, understand, and use the information to maintain and promote a state of good health [10]. To be health literate means placing one’s own health and that of one’s family into the specific context, understanding which factors are influencing it, and knowing how to address them. People with adequate level of HL have the ability to take responsibility for one’s own health as well as one’s family health [11].

Caregiver HL seems to have a direct effect on the number of hospitalizations, the hospital length of stay, as well as on the number of accesses to the emergency room of the assisted people, and the resulting costs [12, 13]. In fact, a low level of caregivers’ HL is associated with greater difficulty in interpreting medical prescriptions, reduced adherence to therapy and healthy behaviors, inability to recognize adverse drug reactions and possible therapeutic failures [14].

All this evidence should lead to systematically assess the HL of older adults’ caregivers. In the United States, 52% of caregivers of patients with heart failure and 31% of caregivers of Hispano-American ethnicity do not have an adequate level of HL to meet the specific needs of older care recipients [15, 16]. A study conducted in Brazil showed that 27% of the caregivers had inadequate HL [17]. A recent study conducted in Tuscany showed a high likelihood or possibility of inadequate HL in more than 70% of formal paid caregivers of not self-supporting older adults [18]. Limited information is available regarding caregivers’ HL in the dementia setting and it is not known how it is associated with patients’ health status.

A Chinese project reported that only 16.5% of the caregivers of patients with cognitive impairment had adequate level of HL [19], while a study conducted in Norway showed that almost two-thirds of the caregivers were at an advanced level of HL and only 9% reported inadequate or marginal levels of HL [20]. Another study found that 38% of informal caregivers of adults with memory loss presented limited HL [21].

The primary aim of this study was to measure the prevalence of inadequate HL in a sample of informal family caregivers of older adults with dementia. As a secondary aim, we assessed the relationship of HL with characteristics of caregivers and persons with dementia.

## Methods

This research is part of a larger one, aimed at describing the characteristics of the caregivers of older people with cognitive impairment, with a focus on their HL (cross-sectional phase), and to assess the impact of their HL skills on the health outcomes of persons with dementia (longitudinal phase). The study was approved by the Ethics Committee of the Local Health Authority which is responsible for territorial jurisdiction (Area Vasta Toscana Centro, CEAV 13592_oss) and was conducted according to the Helsinki Declaration. Our study aimed at enrolling either informal or paid caregivers. In this paper, due to the low number of formal caregivers enrolled, only data regarding the familial dyads (person with dementia and family caregiver) collected in the cross-sectional phase were analyzed.

### Study design

Persons with dementia and their caregivers were enrolled in the outpatient geriatric memory clinic of the Careggi Teaching Hospital (Florence, Italy). During the recruitment phase, study participation was systematically proposed to the eligible people, and written permission was asked to both the caregiver and the assisted senior, whenever the person with dementia was able to express his/her willingness to participate. In case of severe dementia, a waiver of informed consent was allowed by the Ethics Committee.

As inclusion criterion, patients were aged over 65 years, were referred to the geriatric memory clinic, had received a dementia diagnosis according to National Institute on Aging criteria [22] and had a score 4 + at the Global Deterioration Scale (GDS) [23]. Caregivers were eligible if they took care of the subject for at least 4 h per week. Subjects living in nursing home or without a knowledgeable caregiver present at the outpatient visit were excluded.

The recruitment started in August 2018 and ended in March 2021, with an interruption since March 2020 until October 2020 due to COVID-19 pandemic.

The sample size of the study was calculated considering the primary aim. Using previous data collected among caregivers of older adults in Tuscany as the expected value^17^, considering a confidence interval of 95% and a margin of error equal to 0.05, the sample size was established as 180 caregivers. Subjects were consecutively screened for inclusion criteria during 1–2 days per week, according to study personnel availability, until the sample size was reached.

A total of 180 caregivers were enrolled, including 174 informal caregivers and 6 formal caregivers. For the purposes of the analyses presented in this paper, due to the low number of formal caregivers enrolled, only informal caregiver-elderly dyads were considered. The six formal caregivers were excluded to reduce the heterogeneity of the sample and improve the generalizability of the results. Due to the expected demographic, social and health status difference of offspring and spouse caregivers, the features of the two groups were compared.

### Assessment of caregivers

Caregivers’ data were collected using a questionnaire, administered by members of the research team. The questionnaire included all the scales and questions described below. All the scales or the questions were administered by an interviewer, except for the Test of Functional Health Literacy in Adults (S-TOHFLA), that was self-administered.

The following data were collected: socio-demographic information (date of birth, gender, nationality, mother tongue, level of comprehension of Italian language whether from abroad, years of education), cohabitation and kinship with the person with dementia, number of weekly hours devoted to assistance, and level of health literacy.

A screening of caregiver’s cognitive impairment was performed by means of the Mini-Cog, a 3-min screening tool which assesses performance on two cognitive tasks: three-item word recall and clock drawing test. It allows to suspect a cognitive impairment if the subject is able to recall only one or two of the three words and is not able to correctly draw the clock, or is not able to recall any of the three words irrespectively of the clock performance [24].

HL was measured using two different objective tools: the Newest Vital Sign (NVS) and the short form of the S-TOFHLA (Table 1) [25]. The NVS is validated in many languages including Italian [26]; it was developed to measure HL in clinical settings and then applied in other contexts, such as in population-based studies and among paid caregivers [18, 27–31]. The S-TOFHLA has initially been developed for clinical settings, mainly to detect persons with low HL and has been further used to measure HL among caregivers of older adults [32]. The Italian version of the S-TOFHLA was developed after a strong cultural-adaptation process [33].

**Table 1:** Measurement tools used for the assessment of caregivers’ health literacy

Characteristics	Newest Vital Sign (NVS)^22^	Short form of Test of Functional Health Literacy in Adults (S-TOFHLA)^22^
Skills assessed	Literacy, comprehension, numeracy, application/function, evaluation skills	Literacy, comprehension, numeracy evaluation skills
N items	7	40
Time to be administered	5 min	12 min
Administration mode	Face-to-face interview	Self-administered (paper and pencil) with the support of the interviewer
Description	It is based on the information presented on a nutritional label, printed on a separated sheet, that the caregiver consults during the test administration, and on 7 questions whose answers have to be deduced from the nutritional label	It is divided into two sections. The first one includes four verbal numeracy questions, requiring interpretation of medication instructions, medical test results and an appointment date. The second section includes 36 close-type items regarding the preparation for a radiological examination (passage A) and a health administrative rights management section (passage B)
Scoring and classification	The final score is calculated as the number of correct answers (from 0 to 6) and classifies the subjects into one of three categories: high likelihood of limited HL (score: 0–1); possibility of limited HL (score: 2–3); and adequate HL (score: 4–6)	To each item of the numeracy section, a score of 7 was assessed for correct answer, giving a total of a maximum potential score of 28. For the second section, a score of 2 was assigned to each correct answer, reaching a maximum potential score of 72. The final score was calculated by summing the partial scores of the two sections, and ranged from 0 to 100. According to the final score, three levels of HL were identified: inadequate (0–53), marginal (54–66) and adequate (67–100)

### Clinical features of persons with dementia

For each person with dementia, a comprehensive geriatric assessment was performed, including demographics, global functional status, and cognitive impairment. The functional status was assessed with the Katz’s Activities of Daily Living (ADL) [34] and Lawton’s Instrumental Activities of Daily Living (IADL) scales [35]. Specifically, the ADL (range of the score: 0–6; greater value indicating better function) assesses the person’s abilities to perform six basic activities of daily life, while the IADL (range of the score: 0–8) concerns the ability to use the tools necessary to execute some fundamental daily activities, with higher scores indicating better performance. To assess the cognitive impairment, Mini-Mental State Examination was used, including correction for Italian norms of age and education (MMSEc, range of the score: 0–30; higher scores indicating better performance) [36]. Stage of cognitive impairment was assessed by means of the Global Deterioration Scale (GDS) [23], which has a range between 1 and 7, where 1 indicates a normal cognitive level while 7 severe dementia. Finally, the analysis of behavioral and psychological symptoms associated with dementia was assessed with the Neuro-psychiatric Inventory (NPI) [37, 38], a 12 items scale that rates the frequency and severity of behavioral symptoms during last 4 weeks according to caregivers’ report. If a symptom is reported, a score is calculated multiplying its frequency (1 to 4) by its severity (1–3), with the total scoring being represented by the sum of the domain scores.

### Statistical analysis

All analyses were conducted using R (version 4.0, GNU GLPv2 license). Distribution normality was assessed using the Shapiro–Wilk test. Normal continuous or discrete variables were presented as mean and standard deviation (SD). Asymmetrical continuous or discrete variables were presented as median and interquartile range (IQR). Categorical variables were presented as percentages. The characteristics of spouse and offspring caregivers were compared using the Kruskal Wallis test.

For univariate and multivariate analyses, discrete and continuous variables were dichotomized using the median as cutoff point (age of the caregiver: 59 years; schooling of the caregiver: 13 years; MMSEc: 16; GDS score: 5; NPI score: 11; ADL score: 3). For HL levels according to NVS, “adequate HL” and “possibility of limited HL” were grouped and compared with “high likelihood of limited HL”. For HL levels according to S-TOFHLA, “marginal” and “adequate HL” were grouped and compared with “inadequate HL”. The Mini-Cog was considered positive if the score was 0, 1 or 2 while negative for score higher than 2.

Simple and multiple logistic regression analyses were used to evaluate caregiver’s characteristics (age, gender, years of schooling, relationship to patient with dementia) as independent variables and the HL levels according to NVS and S-TOHFLA, respectively, as dependent variable. The same analyses were used to examine the independent association between caregiver’s (independent variables: age, gender, years of schooling, relationship to person with dementia, NVS, Mini-Cog) and assisted people characteristics (dependent variables: cognitive decline measured using MMSEc, and GDS; behavioral impairment of symptoms using the NPI; functional status using ADL). The multiple logistic regression analysis was performed including only dyads in which the caregivers were spouses or offspring, excluding more distant relatives and friends, due to the limited number of caregivers in this group (*N* = 9). The models used to investigate the association with MMSEc, NPI and ADL were adjusted for both the characteristics of the caregivers and the characteristics of the elderly assisted. The model used to assess the association with GDS was adjusted only for the characteristics of the caregiver, since the GDS scale already evaluates various areas concerning cognition, function and behavioral symptoms.

Multicollinearity was assessed using variance inflation factor (VIF). For all the analyses, a *p* value of 0.05 was considered as significant.

## Results

### Description of the sample

A total of 174 person with dementia/caregiver dyads were enrolled in the study (Table 2).

**Table 2:** Demographic and clinical characteristics of caregivers and older adults (*N* = 174)

Subgroups	Variables		Median (IQR)	*N* (%)	NA
Caregivers					
	Age (years)		59 (55–71)	–	1
		≤ 65	–	119 (69%)	
		> 65	–	54 (31%)	
	Gender	Female	–	124 (71%)	0
		Male	–	50 (29%)	
	Relationship to care recipient	Spouse	–	52 (30%)	1
		Son/daughter	–	112 (65%)	
		Other	–	9 (5%)	
	Cohabitation with care recipient	Yes	–	77 (44%)	2
		No	–	96 (56%)	
	Caregiving hours per week	30 (12–168)	–	10	
	Years of schooling	13 (8–14)	–	3	
	NVS	Score	4 (2–6)	–	3
		High likelihood of limited HL	–	36 (20%)	
		Possibility of limited HL	–	44 (25%)	
		Adequate HL	–	97 (55%)	
	S-TOFHLA	Score	96 (89–100)	–	20
		Inadequate HL	–	13 (8%)	
		Adequate or marginal HL	–	145 (92%)	
	Mini-Cog	Score	5 (3–5)	–	2
		Positive	–	15 (9%)	
		Negative	–	157 (91%)	
Older adults	Age (years)	85 (79–88)	–	0	
	Gender	Female	–	126 (72%)	0
		Male	–	48 (28%)	
	Number of falls	0	–	117 (68%)	3
		≥ 1	–	54 (31%)	
	MMSEc score	16.4 (10.4–20.0)	–	4	
	NPI score	11 (6–18)	–	2	
	GDS	Score	5 (4–6)	–	1
		Stage 4	–	72 (41.6%)	
		Stage 5	–	46 (26.6%)	
		Stage 6	–	49 (28.3%)	
		Stage 7	–	6 (3.5%)	
	Functional state	ADL	3 (2–5)	–	0
		IADL	1 (0–3)	–	2

*MMSEc* mini mental state examination corrected according to age and education, *NVS* newest vital sign, *S-TOFHLA* short form of the test of functional health literacy in adults, *NPI* neuro-psychiatrics inventory, *GDS* global deterioration Scale, *ADL* activities of daily living, *IADL* instrumental activities of daily living, *NA* not available

The median age was 59 (IQR 55–71) years for caregivers and 85 (IQR 79–88) years for persons with dementia. Most of the caregivers were sons/daughters (65%) or spouses (30%). Seventy-one percent of caregivers and 72% of persons with dementia were female. The caregiver lived with the assisted person in 44% of cases. Most of the sons/daughters (56%) did not cohabit with the care recipient. Caregivers reported spending a median of 30 (IQR 12–168) hours/week caring for the assisted relative. Most of the spouse caregivers (74%) provided care for ≥ 140 h/week.

Almost all the caregivers were Italian (98%). The mean level of education was 12 (± 5) years for caregivers and 7 (± 4) years for persons with dementia. According to the NVS, high likelihood of limited HL, possibility of limited HL and adequate HL were found in 20%, 25% and 55% of the sample, respectively, and 3 subjects did not complete the questionnaire. Considering the S-TOFHLA, 20 subjects did not complete the questionnaire and, among completers, 92% of caregivers had adequate HL, 8% had marginal or inadequate HL. The proportion of caregivers with possible cognitive impairment according to the Mini-Cog was 9%, and higher (22%) among spouse caregivers (p = 0.001). Caregivers with possible cognitive impairment (i.e., with positive Mini-Cog) had lower NVS and S-TOFHLA scores compared to those with a negative test (NVS: median = 2 vs 4; S-TOFHLA: median = 50 vs 96).

The characteristics of spouse caregivers were significantly different from those of offspring caregivers. The spouses were older (median = 76 vs 57, *p* < 0.001), had a lower level of HL (NVS: median = 2 vs 4, *p* < 0.001; S-TOFHLA: median = 88 vs 98, *p* < 0.001), had a lower level of education (median = 8 vs 13, *p* < 0.001), and provided more hours of care per week (median = 168 vs 20, *p* < 0.001). Moreover, among offspring, two (2%) had marginal or inadequate HL on the S-TOFHLA, and 42 (36%) possibility or high likelihood of inadequate HL according to the NVS.

### Predictors of low HL

Table 3 shows the results of simple and multiple logistic regression analysis performed to evaluate caregivers’ features associated with low HL. Older age, female gender, and a lower level of education (< 13 years), but not kinship with the patient, were independently associated with lower levels of HL (age: ORa = 1.09, 95 CIs = 1.02–1.19, *p* = 0.02; gender: ORa = 3.28, 95% CI = 1.10–11.1, *p* = 0.04; years of education: ORa = 4.68, 95% CI = 1.73–13.6, *p* = 0.003).

**Table 3:** Simple and multiple logistic regression analysis: predictors of caregivers’ health literacy according to Newest Vital Sign (“high likelihood of limited health literacy” vs “adequate or possibility of limited health literacy”)

Variables		Simple logistic regression analysis		Multiple logistic regression analysis	
		OR [95% CI]	*p*	OR [95% CI]	*p*
Age (years)	≤ 59	–	–	–	–
	> 59	1.11 [1.07; 1.15]	< 0.001	1.09 [1.02; 1.19]	0.021
Gender	Male	–	–	–	–
	Female	1.17 [0.52; 2.85]	0.7	3.28 [1.10; 11.1]	0.043
Kinship with care recipient	Spouse	–	–	–	–
	Offspring	0.13 [0.06; 0.30]	< 0.001	0.68 [0.13; 3.80]	0.7
Years of schooling	≥ 13	–	–	–	–
	< 13	9.23 [3.90; 24.6]	< 0.001	4.68 [1.73; 13.6]	0.003

*OR* odds ratio, variance inflation factor < 5

### Caregiver’s HL and health status of persons with dementia

Results of logistic regression analysis regarding the association of NVS scores with health of persons with dementia are shown in Table 4. An inadequate caregiver’s HL was associated with greater severity of cognitive impairment as assessed by MMSEc and GDS, both on the simple logistic regression (respectively, ORa = 2.62, 95% CI = 1.21–5.93, *p* = 0.017; ORa = 3.25, 95% CI = 1.42–8.18, *p* = 0.008) and after controlling for characteristics of caregiver and persons with dementia, (respectively, ORa = 6.58, 95%CI = 2.05–24.6, *p* = 0.003; ORa = 5.61, 95% CI = 1.97–18.1, *p* = 0.002).

**Table 4:** Predictors of greater cognitive deterioration (model 1—MMSEc: score < 16 vs >  = 16) and severity of behavioral and psychological symptoms (model 2—NPI: score <  = 11 vs > 11): simple and multiple logistic regression analysis. The models were adjusted for both the characteristics of the caregivers and the characteristics of the elderly assisted

Models	Variables			Simple logistic regression analysis		Multiple logistic regression analysis	
				OR [95% CI]	*p*	OR [95% CI]	*p*
1.Dependent variable: MMSEc	Caregivers	NVS	Possibility of limited or adequate HL	–	–	–	–
			High likelihood of limited HL	2.62 [1.21; 5.93]	0.017	6.58 [2.05; 24.6]	0.003
		Age (years)	≤ 59	–	–	–	–
			> 59	0.99 [0.97; 1.02]	0.6	1.00 [0.95; 1.07]	0.89
		Gender	Male	–	–	–	–
			Female	0.85 [0.43; 1.70]	0.7	0.39 [0.14; 1.01]	0.06
		Relationship to care recipient	Spouse	–	–	–	–
			Son/daughter	1.81 [0.92; 3.62]	0.087	13.4 [3.05; 73.6]	0.001
		Years of schooling	≥ 13	–	–	–	–
			< 13	1.39 [0.74; 2.64]	0.3	1.59 [0.64; 4.06]	0.32
		Mini-Cog	Negative	–	–	–	–
			positive	2.46 [0.71; 8.54]	0.16	5.40 [1.15; 29.7]	0.04
	Older adults	NPI	≤ 11	–	–	–	–
			> 11	2.39 [1.26; 4.61]	0.054	1.80 [0.81; 4.01]	0.15
		ADL	≥ 3	–	–	–	–
			< 3	3.89 [2.04; 7.61]	0.001	4.92 [2.26; 11.2]	< 0.001
2.Dependent variable: NPI	Caregivers	NVS	Possibility of limited or adequate HL	–	–	–	–
			High likelihood of limited HL	2.42 [1.08; 5.89]	0.039	1.89 [0.66; 5.59]	0.2
		Age (years)	≤ 59	–	–	–	–
			> 59	1.02 [0.99; 1.05]	0.2	0.99 [0.94; 1.04]	0.8
		Gender	Male	–	–	–	–
			Female	0.84 [0.42; 1.65]	0.6	1.16 [0.49; 2.70]	0.7
		Relationship to care recipient	Spouse	–	–	–	–
			Son/daughter	0.79 [0.40; 1.54]	0.5	0.81 [0.23; 2.80]	0.7
		Years of schooling	≥ 13	–	–	–	–
			< 13	1.67 [0.88; 3.24]	0.12	1.45 [0.65; 3.28]	0.4
		Mini-Cog	Negative	–	–	–	–
			Positive	1.65 [0.47; 72]	0.43	1.67 [0.39; 8.85]	0.5
	Older adults	MMSEc	≥ 16	–	–	–	–
			< 16	2.39 [1.25; 4.55]	0.008	1.85 [0.85; 4.09]	0.12
		ADL	≥ 3	–	–	–	–
			< 3	2.05 [1.09; 3.92]	0.027	2.09 [1.00; 4.42]	0.051

*MMSEc* mini mental state examination corrected according to age and education, *NVS* newest vital sign, *NPI* neuro-psychiatrics inventory, *GDS* global deterioration Scale, *ADL* activities of daily living, *OR *odds ratio; variance inflation factor < 5

The relationship with care recipient was also independently associated with cognitive impairment at the MMSEc and the GDS: those assisted by an adult child caregiver presented greater cognitive impairment (respectively, ORa = 13.4, 95% CI = 3.05–73.6, *p* = 0.001; ORa = 3.35, 95% CI = 1.07–11.4, *p* = 0.04) (Tables 4 and 5). The HL was associated with NPI in the univariate analysis (ORa = 2.42, 95% CI = 1.08–5.89, *p* = 0.039), but not in the multiple logistic regression analysis adjusted for MMSEc and ADL (*p* > 0.05). No association was found between HL and ADL scores in both univariate and multivariate analysis (*p* > 0.05). Except for a significant association with MMSEc scores (*p* = 0.04), no association was found between the possible cognitive impairment of the caregivers according to the Mini-Cog and the characteristics of persons with dementia (Table 4).

**Table 5:** Predictors of cognitive deterioration (model 1 – GDS: score < 5 vs >  = 5) and functional status (model 2 – ADL: score < 3 vs >  = 3): simple and multiple logistic regression analysis. The model used to evaluate the association with GDS was adjusted only for the characteristics of the caregiver, since the GDS scale already evaluates various areas concerning cognition, function and behavioral symptoms

Models	Variables			Simple logistic regression analysis		Multiple logistic regression analysis	
				OR [95% CI]	*p*	ORa [95% CI]	*p*
1.Dependent variable: GDS	Caregivers	NVS	Possibility of limited or adequate HL	–	–	–	–
			High likelihood of limited HL	3.25 [1.42; 8.18]	0.008	5.61 [1.97; 18.1]	0.002
		Age (years)	≤ 59	–	–	–	–
			> 59	1.02 [0.99; 1.05]	0,2	1.01 [0.96; 1.06]	0.7
		Gender	Male	–	–	–	–
			Female	0.84 [0.42; 1.65]	0.6	0.6 [0.26; 1.35]	0.2
		Relationship to care recipient	Spouse	–	–	–	–
			Son/daughter	1.04 [0.53; 2.02]	> 0.9	3.35 [1.07; 11.4]	0.043
		Years of schooling	≥ 13	–	–	–	–
			< 13	1.44 [0.76; 2.76]	0.3	0.96 [0.43; 2.15]	> 0.9
		Mini-Cog	Negative	–	–	–	–
			Positive	1.11 [0.34; 3.90]	0.9	0.99 [0.24; 4.13]	> 0.9
2.Dependent variable: ADL	Caregivers	NVS	Possibility of limited or adequate HL	–	–	–	–
			High likelihood of limited HL	0.82 [0.37; 1.76]	0.6	0.53 [0.18; 1.51]	0.2
		Age (years)	≤ 59	–	–	–	–
			> 59	0.99 [0.96; 1.02]	0.5	1.01 [0.96; 1.06]	0.8
		Gender	Male	–	–	–	–
			Female	1.18 [0.60; 2.35]	0.6	1.17 [0.49; 2.81]	0.7
		Relationship to care recipient	Spouse	–	–	–	–
			Son/daughter	1.71 [0.87; 3.40]	0.12	1.06 [0.29; 3.85]	> 0.9
		Years of schooling	≥ 13	–	–	–	–
			< 13	0.90 [0.47; 1.69]	0.7	0.86 [0.37; 1.98]	0.7
		Mini-Cog	Negative	–	–	–	–
			Positive	0.85 [0.24; 2.79]	0.8	0.48 [0.10; 2.03]	0.3
	Older adults	MMSEc	≥ 16	–	–	–	–
			< 16	3.89 [2.04; 7.61]	< 0.001	4.77 [2.23; 10.6]	< 0.001
		NPI	≤ 12	–	–	–	–
			> 12	2.05 [1.09; 3.92]	0.027	2.06 [0.99; 4.34]	0.053

*MMSEc* mini mental state examination corrected according to age and education, *NVS* newest vital sign, *NPI* neuro-psychiatrics inventory, *GDS* global deterioration Scale, *ADL* activities of daily living, *OR *odds ratio; variance inflation factor < 5

Results of the multiple logistic regression analysis performed using the S-TOFHLA classification instead of the NVS ones are reported in the Supplementary Table S1 and S2. Results are similar to those reported in Tables 3 and 4, in which the NVS has been used as the measure of HL, although associations are not significant, possibly due to the low number of subjects with low HL.

## Discussion

In the investigated sample of caregivers, 45% presented a possibility or a high likelihood of inadequate HL on the NVS. The percentage of subjects with inadequate HL was higher among spousal caregivers than among children (NVS: 73% vs. 36%; S-TOFHLA: 38% vs. 2%). Female gender, higher age and lower education emerged as independent predictors of low HL. Person with dementia assisted by caregivers with a high likelihood of limited HL at the NVS presented higher risk of more severe dementia (MMSEc score < 16, GDS score > 4) (Tables 4 and 5).

The only study that measured the HL of informal caregivers of older adults with memory loss using the NVS showed a slight prevalence of subjects with adequate HL [21]. Yet in that study no formal diagnosis of dementia was established. As a comparison, a study carried out using the NVS in a younger sample of congenital heart defects caregivers found an adequate HL in 79% of participants [39]. HL of offspring was similar to that observed in a previous study conducted on a population-based sample in Tuscany, that reported age and education as predictors of HL [40]. HL levels of spouse caregivers, however, were closer to the estimates obtained in a study conducted on formal caregivers (mostly of foreign origin) [18]. According to the multivariate analysis, lower HL of spouse caregivers is explained by higher age and lower education, and this factor should be taken into account to assess its impact both on the assisted seniors’ as well as on their own health [12, 30, 41, 42]. Of notice, spousal caregivers had lower HL levels, cared for more hours per week than offspring caregivers, and among them, a significant number (22%) tested positive on the Mini-Cog.

In the multiple logistic regression analysis, a lower HL of the caregiver emerged as an independent predictor of a more severe stage of dementia. These results are contrasting with what should be the goal for a better care of person with dementia: the most complex cases should be assisted by caregivers with higher levels of HL, more competent and able to appropriately manage health information and all care-related needs.

Few studies have examined the impact of HL on assisted person’s health status, highlighting a trend toward a positive relationship between a low level of HL of caregivers and negative health outcomes for the assisted people. This relationship had already been investigated in samples of diabetic subjects, with heart disease or hospitalized in palliative care units [15, 43, 44]. Other studies have looked for the same association in older people or pediatric subjects [12, 45]. To the best of our knowledge, this is the first study to describe the association between a low level of HL of caregivers of older people with dementia and the severity of cognitive impairment of the assisted person. It can be hypothesized that the need to assist a relative with a greater cognitive impairment may be associated with higher level of stress and, in turn, in a lower ability to mobilize cognitive resources, resulting in a lower performance as assessed by the HL literacy test, which clearly rely on executive functions. Conversely, we might speculate that persons with dementia assisted by caregivers with inadequate HL could have a higher risk of severe cognitive decline due to sub-optimal assistance required. No significant association was found between caregiver’s HL and the behavioral symptoms (NPI) and the functional status (ADL) of the assisted senior.

However, caregivers’ HL was not associated with severity of behavioral symptoms, which typically may be triggered by inadequate care. Indeed, in our study, the functional status (ADL) and the behavioral symptomatology (NPI) of the patients would seem to be unaffected by the caregiver's HL, suggesting that they probably depend primarily by patients’ clinical characteristics instead of caregivers’ skills. These results will be further investigated in the longitudinal phase of the study, which will allow to deepen these relationships.

In our study, intergenerational caregiving (defined as providing care for mother, father, mother-in-law etc.) was associated with worse cognitive performance of caretakers. This association could be explained by the modality of recruitment, which took place in an outpatient setting. Persons with more severe dementia may have been more likely accompanied by their sons or daughters.

If these results were confirmed, public health interventions to improve caregivers' health literacy would be needed, particularly targeted for spouses of elderly people with dementia, who might represent a high-risk population. In this sense, a multidisciplinary approach including both public health and geriatrics perspectives and expertise has to be applied.

Regarding the S-TOFHLA measurements, we observed a “ceiling effect”, as already described by other Authors [46, 47]. In fact, among son/daughter caregivers, only two (2%) had marginal or inadequate HL on the S-TOFHLA, while 42 caregivers (36%) presented possibility or high likelihood of inadequate HL according to the NVS. The practicality of the NVS as a screening tool in the elderly population appears to be limited, when compared with the S-TOFHLA [46]. This observation is confirmed by a study conducted on diabetic patients [48]. However, a study conducted on a sample of younger adults describes a ceiling effect of the S-TOFHLA compared to the NVS and Yuen et al. argue that in the absence of tools that capture the full spectrum of constructs included in HL definitions, NVS may serve as a sensitive discriminator for assessing HL in adult caregivers [47, 49]. On the other hand, compared to NVS, more caregivers did not complete the S-TOFHLA. In most cases, subjects unable to complete the S-TOFHLA, often due to visual impairment, were older (median age: 79 vs 59 years) and had lower HL levels according to NVS (median score of 1 vs 4) than those who completed it. Therefore, we might expect that the prevalence of HL as assessed with S-TOFHLA would have been higher if all caregivers had completed the test.

### Limitations

Despite the trust relationship of patients and their caregivers with the clinic staff limited the percentage of non-adherence to the study, refusals to participate were not recorded, so we cannot exclude that a selection bias occurred. However, the enrollment of the participants was consecutive and unselected.

In our study there was no distinction between newly diagnosed and already diagnosed patients. This could be a limitation; however, this limit could be mitigated by the inclusion criteria considered in the enrollment: in fact only patients with GDS score greater than or equal to 4 and caregivers who assisted patients for more than 4 h a day were enrolled.

In our study no distinction was made between different types of dementia. Furthermore, caregivers were not classified into primary and secondary caregivers.

The study uses a cross-sectional design, so causality cannot be assessed.

The incomplete data on S-TOFHLA is a limitation of the study, as it prevents us to fully appreciate its ability to assess HL in this case-mix. On the other hand, the lower ability of caregivers to complete this instrument might suggest that it is not adequate to assess older caregivers. Indeed, caregivers who did not complete the S-TOFHLA had lower HL levels according to NVS.

Caregivers with possible cognitive impairment according to Mini-Cog were included to avoid the selection of a “supernormal” sample of caregivers. Although we acknowledge that it may have biased the assessment of health literacy, caregivers’ mild cognitive impairment might affect health literacy per se and it should probably be taken into account when assessing caregiving skills.

### Conclusion

In conclusion, the results of this study highlight that the HL level of family caregivers of older adults with dementia is not always adequate, in particular in case of spouse caregivers, due to higher age, lower education and possible cognitive impairment. The significant associations we found in the cross-sectional phase indicate a possible role of HL in predicting some outcomes of the assisted older adults (the lower the HL, the worse the outcomes). According to these results, caregivers’ HL should be included in the comprehensive geriatric assessment, to plan the most appropriate healthcare pathways. Whenever confirmed in longitudinal studies, these results indicate the need of public policies aimed at supporting dementia caregivers, especially older ones, increasing their level of HL.

## Supplementary Information

Below is the link to the electronic supplementary material.

Supplementary file1 (DOCX 23 KB)

## Data Availability

The data sets generated during and/or analyzed during the current study are available from the corresponding author on reasonable request.
