# Supplementary material for: Health literacy of informal caregivers of older adults with dementia: results from a cross-sectional study conducted in Florence (Italy)
Source: Aging Clin Exp Res. 2022 Oct 19;35(1):61–71. doi: 10.1007/s40520-022-02271-0 (PMC9580430; doi:10.1007/s40520-022-02271-0)
Supplement: Supplementary file 1 — Supplementary file1 (DOCX 23 KB) [file 40520_2022_2271_MOESM1_ESM.docx]

**Supplementary table S1.** Multiple logistic regression analysis of predictors for greater cognitive deterioration (model 1 - MMSEc: score <16 vs >=16) and symptoms (model 2 - NPI: score <=11 vs >11). S-TOFHLA score has been included as measure of health literacy.

| ***MODELS*** | ***Variables*** | | | | **OR [95% CI]** | **p** |
| --- | --- | --- | --- | --- | --- | --- |
| **1.Dependent variable: MMSEc** | Caregiver | S-TOFHLA | ≥ 54 | - | | - |
|  |  |  | < 54 | 4.43 [0.72; 35.2] | | 0.13 |
|  |  | Age (years) | ≤ 59 | - | | - |
|  |  |  | > 59 | 1.02 [0.96; 1.08] | | 0.6 |
|  |  | Gender | Male | - | | - |
|  |  |  | Female | 0.35 [0.12; 0.98] | | 0.054 |
|  |  | Relationship to care recipient | Spouse | - | | - |
|  |  |  | Offsprings | 15.7 [3.40; 98.1] | | 0.001 |
|  |  | Years of schooling | ≥ 13 | - | | - |
|  |  |  | < 13 | 1.47 [0.57; 3.93] | | 0.4 |
|  |  | Mini-Cog | negative | - | | - |
|  |  |  | positive | 4.48 [0.72; 32.2] | | 0.11 |
|  | Older adult | NPI | ≤ 12 | - | | - |
|  |  |  | > 12 | 2.10 [0.91; 4.86] | | 0.081 |
|  |  | ADL tot. | ≥ 3 | - | | - |
|  |  |  | < 3 | 3.43 [1.53; 7.91] | | <0.003 |
| **2.Dependent variable: NPI** | Caregiver | S-TOFHLA | ≥ 54 | - | | - |
|  |  |  | < 54 | 3.85 [0.66; 33.6] | | 0.2 |
|  |  | Age | ≤ 59 | - | | - |
|  |  |  | > 59 | 1.00 [0.95; 1.06] | | >0.9 |
|  |  | Gender | Male | - | | - |
|  |  |  | Female | 1.38 [0.56; 3.46] | | 0.5 |
|  |  | Relationship to care recipient | Spouse | - | | - |
|  |  |  | Offsprings | 0.77 [0.19; 2.90] | | 0.7 |
|  |  | Years of schooling | ≥ 13 | - | | - |
|  |  |  | < 13 | 1.28 [0.53, 3.11] | | 0.6 |
|  |  | Mini-Cog | negative | - | | - |
|  |  |  | positive | 0.79 [0.12; 5.01] | | 0.8 |
|  | Older adult | MMSEc | ≥ 16 | - | | - |
|  |  |  | < 16 | 2.08 [0.92; 4.78] | | 0.08 |
|  |  | ADL tot. | ≥ 3 | - | | - |
|  |  |  | < 3 | 2.53 [1.15; 5.67] | | 0.02 |

MMSEc: Mini Mental State Examination corrected according to age and education; S-TOFHLA: Short form of the Test of Functional Health Literacy in Adults; NPI: Neuro-psychiatrics inventory; GDS: Global Deterioration Scale; ADL: Activities of Daily Living; OR= odds ratio; variance inflation factor<5

**Supplementary table S2.** Multiple logistic regression analysis of predictors for cognitive deterioration (model 1 – GDS: score <5 vs >=5) and functional status (model 2 – ADL: score <3 vs >=3). S-TOFHLA score has been included as measure of health literacy.

|  | ***MODELS*** | ***Variables*** | | **ORa [95% CI]** | **p** |
| --- | --- | --- | --- | --- | --- |
| **1.Dependent variable: GDS** | Caregiver | S-TOFHLA | ≥ 54 | - | - |
|  |  |  | < 54 | 5.61 [0.84; 23.7] | 0.1 |
|  |  | Age (years) | ≤ 59 | - | - |
|  |  |  | > 59 | 1.01 [0.96; 1.06] | 0.7 |
|  |  | Gender | Male | - | - |
|  |  |  | Female | 0.58 [0.23; 1.35] | 0.2 |
|  |  | Relationship to care recipient | Spouse | - | - |
|  |  |  | Offsprings | 3.34 [1.04; 11.6] | 0.048 |
|  |  | Years of schooling | ≥ 13 | - | - |
|  |  |  | < 13 | 1.15 [0.50; 2.66] | 0.7 |
|  |  | Mini-Cog | negative | - | - |
|  |  |  | positive | 0.80 [0.15; 4.13] | 0.8 |
| **2.Dependent variable: ADL** | Caregiver | S-TOFHLA | ≥ 54 | - | - |
|  |  |  | < 54 | 0.97 [0.17; 5.91] | >0.9 |
|  |  | Age (years) | ≤ 59 | - | - |
|  |  |  | > 59 | 0.97 [0.92; 1.03] | 0.3 |
|  |  | Gender | Male | - | - |
|  |  |  | Female | 0.75 [0.29; 1.91] | 0.5 |
|  |  | Relationship to care recipient | Spouse | - | - |
|  |  |  | Offsprings | 1.07 [0.26; 4.33] | >0.9 |
|  |  | Years of schooling | ≥ 13 | - | - |
|  |  |  | < 13 | 0.67 [0.26; 1.66] | 0.4 |
|  |  | Mini-Cog | negative | - | - |
|  |  |  | positive | 0.68 [0.11; 3.59] | 0.7 |
|  | Older adult | MMSEc | ≥ 16 | - | - |
|  |  |  | < 16 | 4.06 [1.84; 9.24] | <0.001 |
|  |  | NPI | ≤ 12 | - | - |
|  |  |  | > 12 | 2.53 [1.16; 5.66] | 0.021 |

MMSEc: Mini Mental State Examination corrected according to age and education; S-TOFHLA: Short form of the Test of Functional Health Literacy in Adults; NPI: Neuro-psychiatrics inventory; GDS: Global Deterioration Scale; ADL: Activities of Daily Living; OR= odds ratio; variance inflation factor<5
